# Continuous norming in learning progress monitoring—An example for a test in spelling from grade 2–4

**DOI:** 10.3389/fpsyg.2022.943581

**Published:** 2022-12-16

**Authors:** Michael Schurig, Stefan Blumenthal, Markus Gebhardt

**Affiliations:** ^1^Faculty of Rehabilitation Sciences, TU Dortmund University, Dortmund, Germany; ^2^Faculty of Philosophy, Institute for Elementary Education, University of Rostock, Rostock, Germany; ^3^Faculty of Human Sciences, University of Regensburg, Regensburg, Germany

**Keywords:** learning progress monitoring, curriculum based measurement, continuous norms, primary school, formative assessment, spelling, learning trajectories

## Abstract

One of the main goals of the teacher and the school system as a whole is to close learning gaps and support children with difficulties in learning. The identification of those children as well as the monitoring of their progress in learning is crucial for this task. The derivation of comparative standards that can be applied well in practice is a relevant quality criterion in this context. Continuous normalization is particularly useful for progress monitoring tests that can be conducted at different points in time. Areas that were not available in the normalization sample are extrapolated, closing gaps in applicability due to discontinuity. In Germany, teachers participated in a state-funded research project to formatively measure their children's spelling performance in primary school. Data (*N* = 3000) from grade two to four were scaled, linked and translated into comparative values that can be used in classrooms independently from specific times. The tests meet the requirements of item response models and can be transferred well to continuous norms. However, we recommend using the 10th or 20th percentile as cut-off points for educational measures, as the 5th percentile is not discriminating enough.

## Introduction

In all countries there are children who benefit only slightly or hardly at all from regular instruction in school. International large scale studies (e.g. PISA or PIRLS) show that between 10 and 20% of elementary school children do not acquire the basic skills in reading and mathematics necessary to enter the secondary school (e.g., Hußmann and Schurig, 2019). Children with barely demonstrable learning growth are often referred to as struggling students or students-at-risk.

Research shows that children's learning development varies and that children learn at different rates depending on the classroom, cognitive prerequisites, motivation, and social environment. In Germany 10–30% of a class show little or no improvement in competencies in Mathematics over a school year, while their classmates show moderate or strong measurable learning growth (Salaschek et al., 2014). A closer look at the learning progression in reading and spelling over several years reveals that the gap between high and low achieving students can even widen (DeVries et al., 2018). Lenhard et al. (2017) for example found that reading proficiency levels continue to diverge, especially in the early grades and that the gaps between children's performance remain constant through eighth grade in Germany. Accordingly, Peng et al. (2019) showed that word reading developmental trajectories did not close until a hypothesized performance plateau was reached in the U.S. In a more recent study, Carvalhais et al. (2021) traced the developmental paths at word, sentence, and discourse levels in Portugal. Lower results corresponded to lower academic years between grades 4 and 7 as well as 6 and 9. Discourse-level predictors were identified as the strongest predictors for a written texts' quality in both cohorts while word- and sentence-level predictors only held explanatory power in the younger cohort. This does indicate the need for specific learning difficulties to be signaled in time to appropriately adapt instruction.

Spelling competence is seen as a key qualification in societies. Spelling competence consists of various aspects as punctuation, error sensitivity, correction of spellings and spelling strategies (see KMK, 2005; Jaeuthe et al., 2020). Spelling strategies include both the ability to write words as they are spoken (phonetically) and the consideration of orthographic and morphemic rules. The development of spelling competence is theoretically described as a hierarchically structured competence level model in Germany (see section research questions). Findings in international research show that the acquisition of spelling can be traced back to several components such as L1 (Verhoeven, 2000), linguistic trajectories in word spelling and distinctiveness of cognitive and linguistic trajectories in non-word spelling (Lervåg and Hulme, 2010). Those components therefore have to be addressed in research work.

One of the main goals of the teacher and the school system as a whole is to close learning gaps and support students with difficulties in learning. At the level of the school system, this is labeled as compensatory effects by schools in Germany (e.g., Herrmann et al., 2021). Here, research showed ambiguous results as compensatory effects are found at least as often as so-called Matthew effects where strong students even profit more than students with difficulties in learning (Herrmann et al., 2021). Compensatory effects at the school level are therefore achieved when the school system supports children with learning problems and allows them to catch up with the other students. Current instruction hardly helps these children lagging behind and needs to be changed (Vaughn et al., 2003; Stanat et al., 2017; Fuchs et al., 2021). This leads to the question if there is an international standard for the identification of students-at-risk and students that are in need of individualized education plans. Additionally, the ambitiousness of the support for both groups of students has to be questioned. The short answer is that there are no international quality standards and often national standards are varying by state or region (Brussino, 2020). National frameworks often remain normative and imprecise (Prince et al., 2018). This leads to the question which economic planning of the funds is efficient and how much individual support is affordable for an education system without withholding resources from students without special needs (Brussino, 2020). In particular, the traditional identification and promotion of special educational needs (SEN) in order to provide more resources to children with learning difficulties is criticized for taking too long, being stigmatizing and not being effective enough (Fuchs et al., 2012).

For children with learning difficulties, support systems with multiple levels of support (MTSS) based on the Response-to-Intervention (RTI) approach have proven to be particularly effective (Fuchs and Fuchs, 2006; Keuning et al., 2017; Arias-Gundín and García Llamazares, 2021) and are now being implemented in more and more countries (Björn et al., 2018). The RTI approach focuses on the learning developments of individual students. It addresses the question to what extent the support works to achieve the learning goal (Fuchs and Fuchs, 2006). To answer this question, students' learning trajectories are monitored and evaluated longitudinally. Thus, for the evaluation of current instructional decision making, several measurements, and information are collected during the learning process since only two pre-post measurements with normed school achievement tests are an insufficient data basis for didactic decisions (Fuchs et al., 2012). Subsequently, decisions about possible adjustments in support are made on the basis of the data collected. Vaughn et al. (2003) see a general paradigm shift away from assessment diagnostics to support diagnostics in the use of data on learning development. Currently, multi-level support systems are implemented in the USA, Finland, the Netherlands and in some regions in Germany (Voß et al., 2016; Björn et al., 2018). In the Netherlands a mandatory participation in the assessment of achievement and achievement development enables data-based adaptive design of instruction on a classroom as well as an individual level. Studies at the school level show positive effects in mathematics and spelling and slightly higher effects for learners with difficulties (van Geel et al., 2016; Keuning et al., 2017). For a comprehensive introduction on the evaluation of intervention programs see Souvignier (2020).

The MTSS usually consists of three levels, which are constructed according to support needs between level 1: “little” to level 3: “need for special education support.” Decisions about the level at which students should be supported can be made on the basis of student's scores on screenings, progress monitoring tests and comparison to normalized scores. In summary, this would be called data-based decision-making. Thus, comparison scores are an important benchmark for educational decision-making to determine whether the individual student now needs and can receive more resources (Fuchs et al., 2012).

In this study, comparative scores in spelling were derived using continuous normalization to test the possibility of making gap-free comparisons with a reference group from a federal state Progress Monitoring (PM) platform. In order to understand the ideas for the implementation presented, it is helpful to look at the requirements for PM systems. These are reflected in their quality criteria.

## Quality criteria of progress monitoring in spelling

The idea of PM is to provide feedback on the effect of instructional support and interventions over time using repeated short, but reliable standardized tests (Tindal, 2013; Schurig et al., 2021). PM is a form of formative diagnostics that measures and evaluates learning developments and provides direct feedback to teachers and learners (Gebhardt et al., 2021). The aim of PM is to document learning or behavioral development in a precisely formulated area as accurately as possible and necessary, thus enabling teachers to make fact based instructional or educational decisions. The path to the learning goal and the achievement of the defined goal are measured by means of easily manageable short tests as individual learning developments of the students over time (Hosp et al., 2016). This poses multiple substantial and operational challenges. The identification of characteristics and components of the monitored constructs, in the case of this study spelling as an overall competence, but also individual skills for successfully dealing with individual spelling phenomena and an understanding of their interaction, is required as a basis. Spelling development is determined by multiple factors such as cognitive and linguistic components (Lervåg and Hulme, 2010). Mesquita et al. (2020) investigated the spelling abilities of second, third and fourth graders in European Portuguese and addressed the orthographic complexity categories digraph, contextual consistency, position consistency, consonant cluster, stress mark, inconsistency, and silent letter ‹h›. Differential developmental trajectories per complexity category were found. Kim et al. (2016) found that in Korea learning growth in spelling can be modeled as a function of the orthographic transparency and the differing skill levels of students. Both results indicate that the difficulty of the words to be spelt must be taken into account in the choice of test material. To systematize this difficulty, a review of models of spelling acquisition in German is necessary. In Germany, there is a multitude of models for the development of spelling in primary school. These include models

- Of gradual understanding between the meaningfulness to the lexical order of writing (Brügelmann and Brinkmann, 1994),
- Of the strategies between logographeme (e.g., writing of letters or words from memory) and word spanning (e.g., the orthographically correct composition and choice of linguistic means through orientation on sentences, paragraphs or whole texts; May, 1990),
- Of phases ranging from proto-alphabetic phonetics to correct spelling with few overgeneralizations (Thom, 2003) or
- Of profiles from an alphabetic/phonologic strategy to an orthographic/grammatical strategy (Reber and Kirch, 2013).

But there are strong intersections in the successive levels (even when connoted as steps, strategies, phases or profiles) of competence, with three levels appearing in all models: (1) Not yet phonetically correct spelling including even scribbled characters or single letters. (2) Phonetically correct spelling with spelling corresponding to pronunciation and (3) orthographically correct spellings with spellings that cannot be explained exclusively by the pronunciation (Jaeuthe et al., 2020). Therefore, a hierarchical structure of spelling competence levels is assumed. But often it remains vague how students are assigned to a developmental step and while common mistakes are attributed to levels students are most often not in longitudinal designs (see Jaeuthe et al., 2020).

The tests have to give a reliable and valid measure of change within students as well as an option to compare growth measures between specific groups of students (Anderson et al., 2017). As with other tests, learning progress monitoring instruments need to address main quality criteria of tests: objectivity, reliability and validity (Good and Jefferson, 1998). However, these criteria must apply not only to data points collected once, but moreover to changes in the data over time. Therefore, homogeneity of the measured constructs over time and sensitivity are—besides the calibration of the tests—also quality criteria for learning progress monitoring tests. Progress monitoring tests must be tested for dimensionality and fairness over time and for different subgroups, so the application of Item-response Theory (IRT) or structural equation modeling is recommended (Wilbert and Linnemann, 2011; Schurig et al., 2021). Criteria that relate to the practical application of tests of learning progress assessment have to be considered too. Social comparison is highly relevant when progress monitoring is used to make statements in relation to the individual reference norm. Accordingly, norms for the change of a competence over time are needed (Hosp et al., 2016; Förster et al., 2017).

For repeated short term measurements of a specific domain, multiple parallel tests and equivalent items are needed to prevent memory effects and different substantial domains from confounding the measures. In parallel test forms, item difficulties within tests differ while the measured domain and overall difficulty are constant between forms of the test (i.e., Embretson, 1996). This way, no additional (possibly varying) variables confound the measures and the difficulty. This can be tested by the analysis of the item parameters as well as the functions of growth. Performance-specific, potentially non-linear, growth functions over time that can be shown to be as invariant as possible for subgroups are desirable. This leads to the question of the fairness of the PM.

There are different options to view the fairness of a test, though none has been agreed upon generally (e.g., American Educational Research Association, 2014). The fairness of a test depends on its purpose. One of the main problems in the development of learning progress tests is that each test is supposed to be equally difficult for each observation and that the test has to be fair for all children in the targeted population (Wilbert and Linnemann, 2011; American Educational Research Association, 2014; Klauer, 2014) including students with SEN. Formative tests must also be comparable for each child across different observations, since performance assessment should relate to individual development over time in the specific dimension being assessed. So, tests have to be equally fair for the same students multiple times.

Test fairness between individuals can be defined as the constancy in difficulty of different groups of test takers within time. For academic progress monitoring items, such as items in spelling, there is the problem that exactly the same items may not be used for each measurement for memory effect reasons. This links to the definition of the test's homogeneity of difficulty. Analysis of differential-item-functioning or measurement invariance can be implied to assess the fairness of the test by group-defining traits (e.g., with or without SEN). If this criterion is met, comparative means may be given to support the usability of the test by giving references to comparable test-takers as well as test-takers for which the test may be too easy or too demanding. This directly addresses the sensitivity of tests.

PM can be constructed for short or longer observation periods. Short, sometimes even weekly, intervals require testing that is as sensitive as possible. This might be used to measure the effect of an intervention in a narrowly defined subarea of a competency or skill (Hosp et al., 2016). Tests that measure an entire competency (e.g., spelling) may have different tasks from several sub-areas (e.g., different orthographic difficulties such as the number of graphemes or diphthongs). For such tests, measurements with longer time intervals are, for example, monthly, semi-annually, or annually. While tests with shorter observation periods are mainly used for measuring individual learning development, tests with longer measurement periods are also (but not exclusively) used as screenings and as a comparison between students (as in benchmarks).

In addition to measuring the psychometric quality, this also requires an interpretable normalization of the tests with comparisons to age or grade cohorts; the derivation of norms. The challenge is thus: It has to be ensured that the test in question is sensitive enough to detect (eventually small and slow) change within a specific domain (Kazdin, 2011; Klauer, 2014). This can be achieved by the implementation of appropriate scoring mechanisms to allow for the comparison of means across time. After a scale is established, mean change, if possible comparisons against national or state-wise percentiles and individual change may be assessed to evaluate the sensitivity of a test across time (Hasbrouck and Tindal, 2006). But while standardized psychological tests or repeated summative tests are most often taken in equidistant and fixed intervals, tests in PM that are taken in classroom situations will often be taken when convenient. Test times could be omitted or postponed for educational reasons, individual students could be repeatedly absent due to another intervention, or holiday periods could cause gaps in observation and an effect on learning. Therefore, time-independent comparisons are desirable.

For the evaluation of mean change repeated measures analysis of variance might be applied (e.g., Souvignier et al., 2014). But this is difficult with non-equidistant time intervals. For individual change a function of the observed scores, most often an ordinary least squares regression (Ardoin et al., 2004), can be computed and evaluated. But this does not address the mean slope (growth) of the comparative sample. For the estimation of mean change latent growth models can be applied, so that latent intercepts and means can be addressed separately (Förster et al., 2017). Additionally, all analyses have to assume an (often very easy) function of growth, such as a linear or quadratic assumption, which does not account for systematic variations of the population (Brunn et al., 2022). But the development of students' performance does not necessarily follow linear trajectories (Strathmann and Klauer, 2010; Salaschek et al., 2014; Mesquita et al., 2020). Furthermore, learning trajectories may differ depending on the study period (Christ et al., 2010) and baseline level. This could be addressed by large and highly controlled norm samples with multiple points of measurement each.

But how many points of measurement are needed to estimate a (simple linear) slope and make use of the parameters for individual assessment? The Kratochwill and Levin (2010) and What Works Clearinghouse (2020) offer the assessment that five points of data within each evaluated phase are necessary to reach satisfactory coefficient of determination and according error margins. Christ et al. (2013) suggest six to eight points of data. This trait of a test directly refers to usability.

No test is useful if its results are not put to use. Here, the two main approaches are empowering the teachers using the test and simplifying the design of the test (Deno, 2003). The test's administrators normally are teachers that have received little to none training in the administration and interpretation of diagnostic tools (van Ophuysen, 2010), stressing the need of a feedback design that takes teachers' understanding, interpretation, and use of data for instructional decision-making into account (Espin et al., 2017). In addition, the tests have to be designed in practical and usable ways that are easy to teach and time efficient (Deno, 2003).

No general analytic framework is appropriate in all situations. Interpretations of the results depend on the domain, the difficulty of the test and the intervals between observations (e.g., Hasbrouck and Tindal, 2006). Nevertheless, it is desirable to have comparative values that can be used at any point in the potential study period and that can take into account non-linear developments in several performance levels.

### Norming in learning progress monitoring

Fuchs et al. (2021) differentiate in the application of Data Based Decision Making in (a) its use as universal screening at one point of measurement for the performance level, (b) as interpretation of learning development over several weeks, or (c) as interpretation of instructional utility. For each field of application, standards, benchmarks and norms which are useful have to be developed for the individual instruments. Teachers may use norms to have a comparison in addition to individual data to measure learning progress as a basis for their pedagogical decision and basis for the intensity of pedagogical support (Hasbrouck and Tindal, 2006). The standards often refer to curricular settings and the benchmarks are essentially cut-scores that were determined to predict proficient performance at the end of a year (Hosp et al., 2016). It is assumed that for teachers such categories are easier to interpret than continuous scores, if the categories for those are recognized as benchmarks nationwide (Hosp et al., 2016). But this is not always given (see section research questions). Moreover, a fine-grained interpretation of continuous data adds little value to teachers, as this would imply that for every expression of the norm, there is also a routine to support students (e.g., a tier in a RTI). However, the information on continuous variables is lost in this process of categorization (MacCallum et al., 2002). Therefore, in addition to categories, continuous norms for experts in assessment should also be provided. Whether national benchmarks are formed at all and to what extent this is possible in a federal country is an open question. Regional norms are already a step forward if there is no national agreement (e.g., Shinn, 1998).

Depending on the interpretation of the test, depending on the sample and also depending on the scaling of the test, these standardizations and the possible interpretations differ. For educational decision-making, teachers may interpret both the intercept (indicator for level of competence) and the slope (indicator for learning progression) (Hosp et al., 2016). For this purpose, teachers need not only the child's values but also comparative values from standardized school studies (Danielson and Rosenquist, 2014; Förster et al., 2017). Standardized studies are therefore also necessary for the interpretation of individual as well as collective learning goals and trajectories. Norms should be available over at least four measurement time points and should account for children with specific learning difficulties (Förster et al., 2017).

When external criteria are given on how test scores can be interpreted directly there is no need for reference scores on the population. This is because the evaluation of a test score is then conducted in regard to this threshold. However, the vast majority of psychometric tests aim to classify a test result in relation to a reference population (Lenhard et al., 2019). Norm scores represent the distribution of raw scores in a (hopefully the) designated population. The empirical distribution is therefore assessed by a sample that is as representative as necessary. Norms can be expressed in the form of t-values or percentiles. However, since percentiles do not represent a linear transformation of the raw scores, further computation with percentiles may lead to bias. Therefore, the percentiles are usually transformed into norm scores. These may take the form of stanine scores, z-scores, T-scores. The norming of psychometric tests can be defined as setting up population-based reference scores in order to be able to assess the exceptionality of an individual test result (Lenhard et al., 2019).

Traditional norming has limitations on behalf of the sample size, which tend to become rather large due to separated groups and biased percentiles in the extreme values due to floor effects. Additionally extreme values tend to influence percentiles strongly. Discontinuity gaps are often present in norm tables because of the categorical nature of the way time is metrized (Zachary and Gorsuch, 1985). There are no norm values for the time between the time intervals the norming took place in, limiting their usefulness in PM. In the last place, traditional norming is based upon assumptions on normal distributions of the variables.

Continuous norming is based on modeling rather than distributional assumptions. The term continuous norming refers to the statistical modeling of the development of percentiles as a function of the test and further explanatory variables (e.g., age, gender, grade, SEN). The relation between scores and time is computed by the total sample and not by single groups. This way growth can be addressed very accurately by including more parameters (Zachary and Gorsuch, 1985; Lenhard et al., 2018; Voncken et al., 2019). Continuous norms may be calculated by polynomial regression for normally distributed variables (Zachary and Gorsuch, 1985), other assumed distributions (Voncken et al., 2019) or even without distributional assumptions (Lenhard et al., 2018). For a comprehensive summary on continuous norms see Lenhard et al. (2018). A summary of the steps for the derivation of continuous norms without prior assumptions is given in Lenhard et al. (2019). These can be described briefly: (a) Subsamples are created. (b) If a continuous explanatory variable (e.g., age) is used, categorical groups (e.g., age intervals) are generated. (c) For each case position percentiles are identified. (d) For every explanatory variable and every position of each case in a subsample, power and their products are computed. (e) A stepwise regression analysis is done using the powers and their products to predict the empirical raw score. (f) The Taylor polynomial function is used to predict the raw score based on the explanatory variable(s). (g) The rank is identified with the significant variables from the stepwise regression analysis. Using the identified Taylor polynomial function either norm tables can be generated or norm values can be derived directly based on the measured raw score and age.

Continuous norming is especially relevant for tests where results have to be assessed in regard to age or grade and if the test will be performed at variable times. The potential advantages of continuous norms are therefore the lack of gaps within an age range, a fine age gradation and the extrapolation into ranges that were not available in the sample. Additionally, the required sample size is strongly reduced. In summary, this suggests that continuous norms and standards could be of great importance for the derivation of comparative values in PM procedures. To our knowledge, however, this has not yet been done.

### Research questions

The measuring of spelling skills in progress monitoring is usually done by using a robust indicator approach in both primary and secondary schools. The research focuses on identifying appropriate tasks and assessment options. This is because the wide variability of errors in spelling (e.g., capitalization, punctuation, grammar, inflections as well as sequencing) makes it difficult to determine a proxy indicator. In a systematic review by McMaster and Espin (2007) the correct word sequences and the difference of correct minus incorrect word sequences are the most appropriate indicators across all grades. Nevertheless, the number of correctly written words is most commonly used in school practice because this index is reliable and very easy to evaluate (McMaster and Espin, 2007). Strathmann and Klauer were the first to publish a proposal for a German-language learning progress test to measure spelling competences (Strathmann et al., 2010). This is a pragmatic dictation test that measures students' transcription skills with the number of correct words as a robust indicator. The spelling of words is assessed according to the categories right or wrong, but it is very easy to design multiple parallel forms of tests that are necessary for progress monitoring. To generate the items, the test authors first created a basic vocabulary (Strathmann and Klauer, 2010).

In contrast to the USA, there are no benchmarks or standards for PM tests in Germany. In Germany, the federal structure of the education system complicates or even forbids the use of national standards for progress monitoring, as both curricular content, student support and school types differ significantly between the states (Brussino, 2020). Even basic vocabularies (e.g., the set of words in a language necessary to understand any text in a given language at a given stage of development) differ between states due to dialects (e.g., language varieties). The use of basic vocabulary is an important didactic approach for the acquisition of spelling skills at school in Germany. Such approaches were already used in the GDR (Riehme, 1987), and later also in West German states (Sennlaub, 1985; Naumann, 1987). The reason given for this was the partly limited regularity in German orthography (Brinkmann and Brügelmann, 2014). Almost all the federal states in Germany have recently developed state-specific basic vocabularies. In total, 1915 words are explicitly listed in the vocabularies of the federal states. However, only 8 words are listed in all. A large proportion of 724 words are listed only in one of the basic vocabularies. For a summary, see Blumenthal and Blumenthal (Blumenthal and Blumenthal, 2020).

Although there are three internet platforms that offer scientifically designed and tested instruments for progress monitoring spelling competencies at the moment (Blumenthal et al., 2022), the use or application is still rather unknown in practice and not recommended by the state. In Germany, teachers can use the state-funded research project lernlinie (learning line^1^) to formatively measure their children's spelling performance. The question arises as to whether the available data can be scaled, linked across grades and translated into comparative values that allow individual students to be placed within percentiles. From these considerations, the following research questions were derived:

- Are the test forms IRT scalable and are the reliability values of the person parameters in an acceptable range?
- How strong are the correlations between the person parameters between the points of measurement?
- Is the data sufficient to derive interpretable and meaningful continuous norms?
- What threshold values can be used for the identification of a risk group?
- In a first step, the results of the individual tests are presented and the fit to the Rasch model is demonstrated. In the second step, the norms are formed across the grades using continuous norms.

## Methods

### Sample and design

The longitudinal study includes 3,000 children from second to fourth grade whose spelling performance was assessed at the beginning of the school year and in the middle of the school year. The scoring of the test was done along the categories of right and wrong. The Internet platform www.lernlinie.de offers free screenings and tests that are appropriate to measure progress over time as a print version under free license for all. However, the use of the platform with the automatic evaluation is possible only for teachers of the federal state funding it. This also meant that data protection guidelines of the state were made applicable and relevant background characteristics such as SEN could not be included by the researchers. In the state in question, this information must remain in the schools if guardians have not explicitly released it. This permission was not obtained for the project. In the data analysis, the user data of this platform are evaluated in the spelling tests from second to fourth grade level. Registration and use of the platform are free of charge and voluntary. Teachers can then download the tests as a copy template for a paper-pen test. The teachers enter the students' entries into the database. Then they are analyzed automatically and children's performance are estimated. By now the following normative cut-off points are given: “well below average” for percentile rank <10, “below average” for percentile rank < 25, “average” for percentile rank < 75, “above average” for percentile rank < 90, “well above average” for percentile rank > 90.

The analyses presented here used student data deposited in the database over the 2018/19–2020/21 school years. Tests were administered at 6-month intervals, at the beginning and middle of each school year. The students were distributed among 51 schools from rural or small-town areas. The gender ratio proved to be approximately balanced. General participation was voluntary and schools were free to decide how many and which test dates they participated in. Information on the distribution of students across grade levels can be found in Table 1, the overlap between the participations is given in Table 2.

**Table 1:** Students by grade and gender.

	**Grade and test within the grade**					
	**Grade 2**		**Grade 3**		**Grade 4**	
	**t1**	**t2**	**t1**	**t2**	**t1**	**t2**
Boys	604	324	688	282	561	238
Girls	585	312	589	232	480	211
Total	1,189	636	1,277	514	1,041	449

**Table 2:** Overlap between grades.

	**Number of participations**						
	**6 times**	**5 times**	**4 times**	**3 times**	**2 times**	**1 time**	**Total**
Boys	8	39	74	201	296	963	1,581
Girls	10	34	51	195	261	868	1,419
Total	18	73	125	396	557	1,831	3,000

### Instruments

The Reiner test concept records the spelling performance of elementary school children every 6 months at the middle and end of the school year. The test was developed for lernlinie (see Blumenthal, 2022). The test consists of cloze texts (Taylor, 1953) that are dictated by the teacher and for which the target words are to be written down by the children. For the test construction different German textbooks were analyzed. However, the individual spelling phenomena vary in the textbooks, in terms of when they first appear, by up to 3 years (cf. Diehl et al., 2020). Vocabulary and its scope also vary (cf. Voß and Blumenthal, 2020). For the construction of the item pools, the models for reading acquisition in German (Gasteiger-Klicpera and Klicpera, 2005), models for spelling (Reber and Kirch, 2013), and the recommendations of the Standing Conference of the Ministers of Education and Cultural Affairs of the federal states in the Republic of Germany (KMK) for the subject German (KMK, 2005), as well as contents of selected language books for elementary school were used.

The item selection was therefore based on the following criteria:

- Items correspond to the verbal vocabulary of children between six and ten years of age
- Items follow recommendations of education ministries
- Items correspond to the vocabulary of relevant textbooks
- Items represent different levels of difficulty

The recommendations of education ministries means the reference to the intersections of the multiple German basic vocabularies. From several vocabularies, 808 relevant words for elementary school were chosen. From these, a test pool for each grade level was created according to the rules in Table 3. The item pools overlap and due to a linkable multi-matrix design (Mislevy et al., 1992). In multi-matrix designs alternate test forms are created with items from an item pool. For the Reiner Test this resulted in two different forms of the same test (e.g., the same item pool) per grade level. All tests are therefore linked by anchor items. Anchor items are the items that are used in more than one test form to link the results. A total of 275 of the words were used for linking, 98 across grade levels and 177 within grade levels. Attention was paid to a distribution of spelling phenomena to be observed, so that a spread of item difficulties across the anchor items can be assumed (Blumenthal and Blumenthal, 2020). Thus, in grade 1, especially (but not exclusively) phonetic short words were used; in grade 2, mainly phonetic complex or frequent words as well as words with multiple consonants; in grade 3, words with double consonants, compound nouns, the extension *h* or the consonant compounds *ck* or *tz*; in grade 4, words with double vowels, the consonant compound *chs*, adjectives ending in *-ig* or foreign words.

**Table 3:** Structure of the reiner tests.

**Grade 2**	**Grade 3**	**Grade 4**
1. Phonetic words with 3-4 graphemes 2. Phonetic words with 5-8 graphemes 3. Special/difficult words (diphthongs, umlauts, words with v) 4. Words with double consonant 5. Words with [ck]	1. Common words 2. Phonetic words with 2–3 graphemes 3. Phonetic words with 4–7 graphemes 4. Words with [ie] 5. Words with [ß] 6. Words with [qu] 7. Words with [ck] 8. Words with [v] at the beginning of the word 9. Words with umlaut [ä], [ö], [ü] 10. Words with consonant doubling ll, tt, nn, mm 11. Words with multiple consonants (e.g. nst) 12. Words with stretching h 13. Word combinations	1. Common words 2. Phonetic words without restriction of the number of graphemes 3. Words with diphthongs au, ei 4. Words with [ie] 5. Words with [ß] 6. Words with [x] 7. Words with [tz] 8. Words with [ck] 9. Words with extensions [üh], [ieh], etc. 10. Words with consonant doubling ll, tt, ff, ss 11. Words ending in -ig, -lich, -ung, -heit, -keit 12. Words with prefixes be-, ge-, ent-, ver-, vor- 13. Words ending with [chs], [ks] 14. Words with a double vowel 15. Words with [qu] 16. Words with stretching h 17. Words with pronoun hardening 18. Words with [v] at the beginning of a word 19. Words with vowel derivatives to umlaut [a-ä], [u-ü] 20. Word combinations 21. Foreign word
Examples (Grade 2 Test 2)	Examples (Grade 3 Test 2)	Examples (Grade 4 Test 2)
Wo [where] vom [from] Euro [Euro] Lasso [Lasso] Körper [Body]	dem [the (*dative*)] Lied [Song] springst [*you are* jumping] Blätter [Leaves] Geburtstag [Birthday]	Glück [Luck] gießen [*we are* casting] Frühling [Spring] ängstlich [anxious] unglaublich [unbelievable]

The tests were piloted and a main study with *N* = 4091 children in 192 first to fourth grades and 24 schools showed fit to a unidimensional Rasch model (Voß and Sikora, 2017). The levels of difficulty were chosen in accordance to Embretson (1996) in order to cover a full range of abilities and thus to enable the location of the person parameters against the background of the differing item difficulties. Words were chosen by the item parameters (Blumenthal and Blumenthal, 2020) to represent easy, medium and difficult words. From a psychometric point of view, 732 words could be identified as suitable for assessing spelling competencies from the grade level 2–4.

The formal design of the spelling tests was guided by economic and pragmatic factors that an inclusive school setting entails (Hosp et al., 2016). For example, they were to be feasible as group procedures in a class setting, the test was not to last longer than 15 min and they were to include tasks that were close to instruction, such as simple word dictations with word counts that depended on the grade level and on whether the test was taken at the beginning or in the middle of the school year (grade 2: first test 24 items and second test 36 items, grade 3: first test 36 items and second test 48 items, grade 4: first test 48 items and second test 60 items). All target words were embedded in narrative texts around the identification figure (a pig named Reiner) that were appealing to children in order to a) increase motivation to complete the tests and b) generate content contexts for semantically ambiguous words. It is assumed that spellers with difficulties might use context clues to their advantage (Taylor, 1953; Ehri, 2005).

The complexity of the texts was determined using the LIX index (Lenhard and Lenhard, 2014). The LIX index accounts for surface features of a text (number of words, word length, proportion of long words, and sentence length) and thus forms an indicator for assessing its difficulty or ease. The LIX is the sum of the average sentence length of a text and the percentage of long words (more than six letters). Care was taken to ensure that the LIX values for the text templates were below 40 and could thus be assumed to be suitable for children and adolescents.

Initial analysis of the psychometric adequacy of the developed tests revealed high reliabilities in the range between .90 ≤ α ≤ 0.96. Correlations with convergent procedures (spelling test Hamburger Schreib-Probe 1-10; May et al., 2019) vary between *r* = 0.69 (*N* = 56) and *r* = 0.82 (*N* = 177) and testify to the validity of the instruments. Further evidence of the psychometric quality of the tests was determined in the present study. In Table 4 the descriptive values as well as the accuracy of the tests within the grades are given.

**Table 4:** Descriptive values of the reiner tests.

**Grade**	**Timepoint**	***N***	**Mean**	**Median**	***SE***	***SD***	**Min**.	**Max**.	**Missing**	**Perc. Acc**.	***SD***
2	1	1,189	13.1	13	0.17	5.87	0	24	0	0.55	0.24
2	2	636	20.8	21	0.35	8.71	0	36	0	0.58	0.24
3	1	1,277	23.2	24	0.22	7.99	0	36	0	0.64	0.22
3	2	514	29.7	31	0.5	11.3	0	48	0	0.62	0.24
4	1	1,041	32.2	34	0.32	10.4	0	48	0	0.67	0.22
4	2	449	39.8	43	0.67	14.3	3	60	0	0.66	0.24

## Results

The basic psychometric criteria were analyzed by the application of IRT analysis with TAM (Robitzsch et al., 2021) in *R* (R Core Team, 2021). One-parameter logistic models with marginal maximum likelihood estimators were applied. The random effect models were done with lme4 (Bates et al., 2015) and the visualization was done with GAMLj (Gallucci, 2019) in jamovi (The jamovi project, 2022).

There are different approaches to the computation of continuous norms. Parametric approaches are making assumptions on the distributional shape of the raw scores (e.g., *R* Package GAMLSS; Rigby and Stasinopoulos, 2005), non-parametric regression based approaches and semi-parametric approaches address the norms as latent variables (Lenhard et al., 2018; R Package cNORM). The approach within cNORM offers the beneficial characteristic that it does not require any distributional assumptions. Therefore, in most use cases the data can be modeled more precisely than with parametric methods (Lenhard et al., 2019). This is particularly true for small samples as small as < 100 and skewed distributions. The data as well as the code is deposited as an open-access OSF project (Schurig et al., 2021,^2^).

### Scaling

To assess the fit of the models measures of reliability (EAP Reliability), a measure of local independence (the average of absolute values of the adjusted Yen‘s Q3 statistic; Yen, 1984, see Robitzsch et al., 2021) as well as mean Outfit and Infit values are given (Table 5; Wright and Masters, 1982). The necessary criteria were met within each grade with reliabilities exceeding 0.8, the mean Q3 statistics approximating 0 and the mean item fit values approximating 1. It can be assumed that the usage of sum scores is defensible (Rost, 2004).

**Table 5:** Fit statistics of the used measures.

**Grade**	***t***	***n***	**# items**	**EAP Rel**.	**MADaQ3**	***M***	***SD***	***M***	***SD***
						**Outfit**	**Outfit**	**Infit**	**Infit**
2	1	1,189	24	0.88	0.084	0.99	0.14	1.00	0.06
2	2	636	36	0.92	0.048	1.01	0.21	1.00	0.10
3	1	1,277	36	0.90	0.051	0.99	0.19	1.00	0.11
3	2	514	48	0.93	0.046	1.04	0.37	1.00	0.12
4	1	1,041	48	0.92	0.043	0.99	0.19	1.00	0.10
4	2	449	60	0.94	0.048	1.01	0.32	1.00	0.12

To address the fairness of the test effects of differential item functioning (DIF) between gender groups were analyzed by using Raju‘s Area method (see Wright and Oshima, 2015) implemented in Snow IRT (Seol, 2022). For this method effects sizes were introduced by Wright and Oshima (2015) with cut-off values between < 1 for neglectable effects and >1.5 for large effects of DIF (Magis et al., 2010).

**Figure 1:**
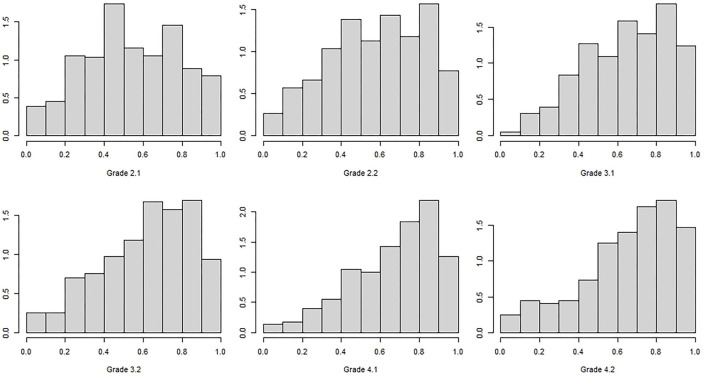
Analyses of the densities of the distributions of the percentage accuracy by Grade.

In Grade 2 time 1 no effects >1 were observed so that a negligible DIF can be assumed (*M*_*abs*_ = 0.37, *SD* = 0.23) and in 2.2 one moderate and one large effect were observed (*M*_*abs*_ = 0.48, *SD* = 0.41). In Grade 3 time 1 one item showed a large effect (*M*_*abs*_ = 0.40, *SD* = 0.44) and in Grade 3 time 2 six items showed moderate and one item showed a large effect (*M*_*abs*_ = 0.54, *SD* = 0.38). In grade 4 time 1, one item showed a large effect (*M*_*abs*_ = 0.49, *SD* = 0.31). In Grade 4 time 2 moderate effects were observed in nine and large effects were observed in six items (*M*_*abs*_ = 0.74, *SD* = 0.60). However, the effects do not have a clear direction which could be interpreted, so that one can assume random and therefore ignorable DIF effects. In the next step the distributions of the sum scores are given. As can be seen in Figure 1 the distribution of the percentage of accuracy is becoming more skewed toward the higher grades. On the y-axis the density is given due to different sample sizes.

When the measures between the points of measurement are correlated, the effects (Spearman rank correlation coefficients; Table 6) range from *r*_*s*_ = 0.51 to 0.85. Roughly speaking, the effects are higher the closer the data points lie to each other in time.

**Table 6:** Spearman correlations between the points of measurement.

		**Grade 2**		**Grade 3**		**Grade 4**	
		**t1**	**t2**	**t1**	**t2**	**t1**	**t2**
Grade 2	t1	1					
	t2	0.74	1				
Grade 3	t1	0.67	0.83	1			
	t2	0.68	0.83	0.79	1		
Grade 4	t1	0.58	0.74	0.67	0.79	1	
	t2	0.51	0.75	0.72	0.85	0.84	1

Only pairwise complete observations were used. All Correlations are significant on a *p* < 0.01 level.

### Random effects modeling

**Figure 2:**
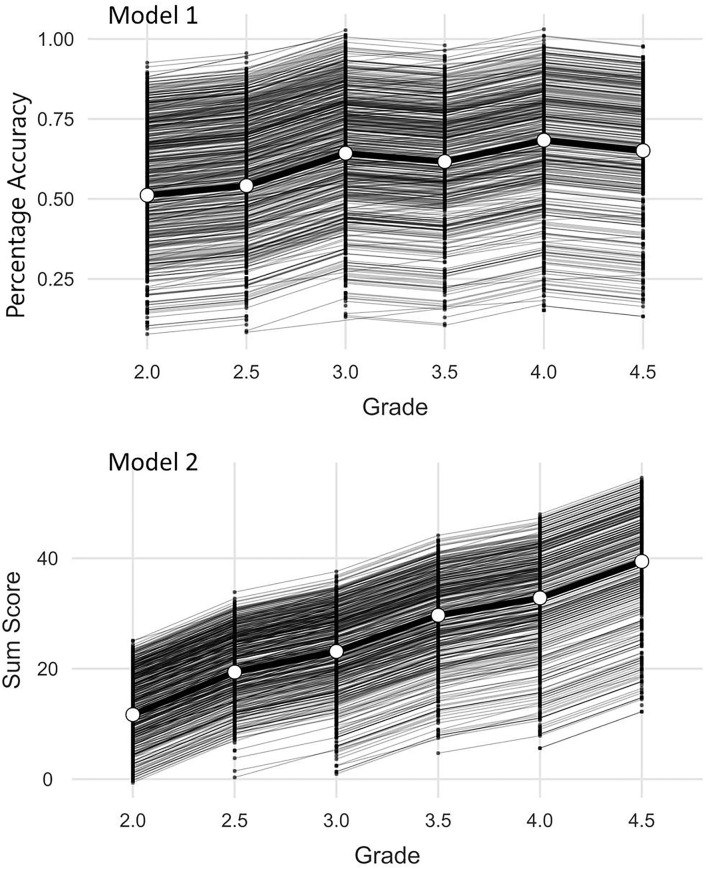
Analyses of random effects models.

In a last step individual growth effects were addressed with a linear mixed model with the scores as random effects within persons and time as a factor (Gallucci, 2019). Here two models were taken into consideration. In the first place (Model 1; Figure 2) the percentage accuracy was analyzed as a random effect. In both models the number of included cases is *n*_*id*_ = 2981. This is the number of students with at least two successive points of measurement and the number of observations within the cases is *n*_*obs*_ = 5087. In the second place (Model 2; Figure 2) the sum of the solved items was analyzed. In Model 1 the stability of the difficulty of the test is addressed. In Model 2 the individual growth in dependence on the increased length of the test (24–60 items) and the increased difficulty of the items (see Instruments) is in the center of interest. The Pearson correlation between the sum scores of all observations and the percentage solved is *r* = 0.822 (*p* < 0.001). Figure 2 (Model 1) indicates that there is no significant floor effect for the difficulty of the tests. The figure of Model 2 shows that there is a compression at the ceiling of the test (the maximum number of items) but that the test also covers low performance and its development, especially from grade 3 onwards.

For both Models fixed effect omnibus tests (Wald) showed significant main effects (Model 1: *F*(5, 2815) = 151, *p* < 0.001; Model 2: *F*(5, 2990) = 1365, *p* < 0.001). The conditional *R*^2^ (variance explanation of the model) in Model 1 is 0.79 and in Model 2 it is 0.86. The marginal *R*^2^ (variance explanation by time alone) is 0.07 in Model 1 and 0.47 in Model 2. The intra class correlation (ICC) of the random component (Student ID) is 0.78 in Model 1 and 0.74 in Model 2. This indicates an expected high variance explanation that can be attributed to the student's proficiency. The low marginal *R*^2^ of Model 1 shows that the difficulty of the test does change significantly but only slightly on behalf of the effect sizes over time which is desirable for the successful linking of the measured values. The fixed effects in Model 2 are larger due to the rising ceiling of the test (Table 7).

**Table 7:** Fixed effects parameter estimates.

	**Model 1 (Percentages)**				**Model 2 (Sum scores)**			
		**95% confidence interval**				**95% confidence interval**	
**Names**	**Est**.	**Lower**	**Upper**	***p***	**Est**.	**Lower**	**Upper**	***p***
Intercept	0.61	0.60	0.62	< 0.001	26.02	25.69	26.36	< 0.001
2.5–2.0	0.03	0.02	0.04	< 0.001	7.71	7.16	8.26	< 0.001
3.0–2.0	0.13	0.12	0.14	< 0.001	11.45	10.95	11.95	< 0.001
3.5–2.0	0.11	0.09	0.12	< 0.001	17.99	17.34	18.64	< 0.001
4.0–2.0	0.17	0.16	0.19	< 0.001	21.14	20.55	21.73	< 0.001
4.5–2.0	0.14	0.12	0.16	< 0.001	27.75	27.01	28.49	< 0.001

In summary, it can be stated that the available data are suitable to a sufficient extent to derive standard values. The relevant question for the linked distributions is whether it is possible to cover a sufficiently broad range of abilities to derive percentiles of interest. Since the aim of these percentiles is to identify students who have difficulties in learning, the relevant question is which percentile is chosen to derive learning difficulties. This can be deduced directly from the assumed volume of a tier of the RTI system implemented nationally or regionally. “In a well-designed RTI system, primary prevention should be effective and sufficient for about 80% of the student population” (National Center on Response to Intervention, 2010). In the model project, it was analogously stated that it can be assumed that ~20% of the students are supposed to receive second tier support (secondary prevention) and up to 5% of the students are supposed to receive intensive individual support (Voß et al., 2016). Taking into account the level of error, progress monitoring, which is used for screening and in addition to possible assessment diagnostics to decide on a support tier, should be selective, especially in the lowest quartile for the second tier (25%) and the lowest percentile for the third tier (10%).

### Normalization

Since multiple regression is used to obtain a model which allows for the estimation of normal values the first step is to identify this model. cNorm utilizes the best regression subset approach to do so (James et al., 2013). The approach returns a regression model, which describes the given norm sample as well as possible with a minimal number of predictors (Gary et al., 2021). These are the explanatory variables (e.g., age or grade), the powers as well as the interactions of person location on the spectrum and explanatory variable. After a model is established, a numerical approximation (not an exact calculation) of the norm score in question can be deduced. For the necessary ranking of the person scores in the grade groups the default procedure was chosen. The degree of the polynomial of the regression function was chosen to be quartic. For the normalization all cases (*N* = 3000) were included even though the sample sizes varied strongly. *n* = 1831 students only took part once. *n* = 557 took part two times, *n* = 396 three times and so forth (see Table 2 in section sample and design). *n* = 1419 girls and *n* = 1581 boys took part at all.

For the model validation an adjusted *R*^2^ value can be used. This is the representation of the approximation of the polynomial on the person score, the estimated norm score and in this case the grade variable. The modeling procedure of the Reiner scores from Grade 2 time 1 to grade Grade 4 time 2 reached an adjusted *R*^2^ = 0.991 (which also is the stopping criterion of the Taylor function in cNorm; Lenhard et al., 2018) with five terms and an intercept. The number of terms was cross validated (20% validation sample; see Gary et al., 2021), repeated ten times and with up to ten terms. No substantial improvement in model fit could be achieved by adding more terms.

**Figure 3:**
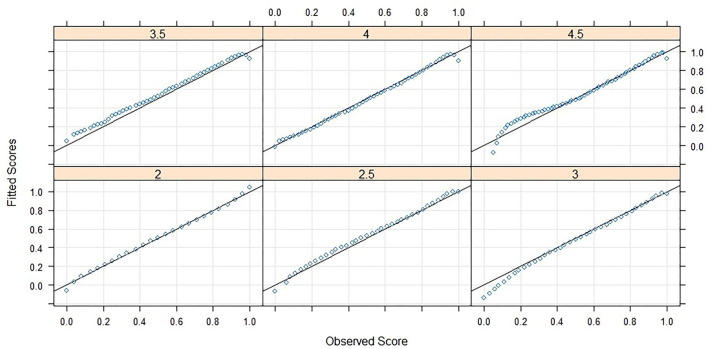
Analyses of observed and fitted raw scores.

Three powers and the related interactions were needed to fit a sufficient model. The root mean square error (*RMSE*), deduced from the difference between the predicted scores and the manifest scores, reaches *RMSE* = 0.0224. When taking into account that the person score in question is a percentage with possible values between 0 and 1 the error is justifiable. In Figure 3 it can be seen that the fit is worse in the area of extreme values especially in the higher grades but in most cases the fitted scores are approximating the observed scores well.

**Figure 4:**
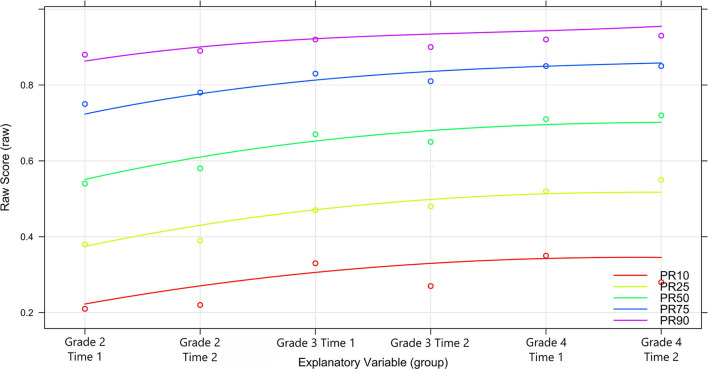
Analyses of percentile curves.

The observed and predicted percentile curves are given in Figure 4. PR stands for percentile rank and the following number the percentile. For example, PR50 describes the 50th percentile. It can be seen that the changes of the test designs (more and more difficult items) are reflected in the observed normal values. A high proximity of the percentile curves to each other indicates poor separability between these curves as can be seen above the 75% percentile. The lower ranks are clearly separable though.

To check which percentiles can be loaded in terms of content, it is possible to inspect the confidence intervals of the norm scores (here T-values) within the measurement time points. This is relevant for the research question on possible threshold values for students-at-risk. However, since these are estimated from the complete population, they reach roughly 10% with a 90% confidence interval, regardless of rank and when controlling for regression to the mean, given the smallest observed reliability of 0.88 [for details on the estimation of the C.I. see Lenhard et al. (2018); see Supplementary Table 1].

With reference to the underlying test, however, it is desirable to achieve a high discriminatory power for the ability range that the test is intended to cover in particular. In the case of the present test, this corresponds in particular to the threshold value necessary to separate the 10th and 25th percentiles or (very) roughly t values between 30 to 40 points. In terms of raw scores, this corresponds to a percentage of solved items between 20 and 30% in the 10th percentile and between 40 and 50% in the 25th percentile. The test is therefore easy enough to include information to separate between ranks in the relevant ability domain.

## Discussion

The tests are clearly scalable and reliable and the person parameters correlate strongly across the times of measurement. The results show that achievement gaps between students in our study generally increase over the years. This finding is in line with Herrmann et al. (2021). This is particularly critical given that the teachers involved in our study received the results from the tests in the sense of a formative assessment. Thus, even despite this information, the students with the most difficulties did not succeed in catching up with the rest of the class. But we do not know to what extent the information was used. The interpretation must take into account that the study was conducted in inclusive schools. Thus, this result is also in line with the research that children with special educational needs differ significantly from the performance of normal students and also fail to catch up with this performance by the end of school (Gebhardt et al., 2015). Even with a comprehensively designed system for high-quality instruction for all students and effective support for at-risk children, it may not be possible to adequately address the needs of all children (Voß et al., 2016). The results of the long-term study of the inclusion model in Rügen (Blumenthal et al., 2019) show that prevalence of special needs has been significantly reduced. However, there is a not inconsiderable proportion of students with extensive difficulties at school for whom long term support must also be offered. This is not only a regional phenomenon, but is also evident in the international context (Fuchs et al., 2014, 2017). Research indicates that 5 to 10% of the student population requires intensive intervention in terms of special education support (O'Connor and Fuchs, 2013).

Normalized scores could be readily derived by the applied procedure. But the question on possible thresholds for the identification of students-at-risk has to be answered in regard to error margins of percentiles. The statistical results show that a representation of the fifth percentile range is associated with too large errors in this study. Therefore, such a cut-off is rather inappropriate for extensive educational decisions based upon the test in question. However, it seems appropriate to consider percentile 10 as the smallest cut-off line. We understand MTSS as a tiered system, in a pragmatic approach. It would be nice to determine the exact level of all learners at all points in time, but that doesn't work without a lot of effort and (very long) tests. Ergo: We stick to an indicator that roughly signals to us that something is wrong and then we take a closer look to initiate and optimize support processes. Ultimately, setting a threshold for student achievement is a normative decision. It could be shown that the present test can support this in the range of the 10th percentile. However, whether this is appropriate or whether individual consideration should be given to the 25th percentile is also a decision that must take into account the performance of a school system. A Smart RTI System as proposed by Fuchs et al. (2012) does not rely on error-free measurement on every level and at every point of measurement. In general, it can be assumed that norm statements for teachers should rather refer to coarser categories (percentile limits 10, 25, 50, 75, 90). These serve data-based decisions in terms of level assignment in an MTSS. Finer gradations (as can readily be found in continuous norms) are associated with higher probabilities of error and add little value here. The classification of students between these thresholds over time, without the need for a fixed time interval for testing, can thus make it possible to classify them in MTSS systems with sufficient certainty for this purpose. It must be clear, however, that a single measurement is not sufficient to map a learning process and that this single measurement cannot be seen as a “substitute” for a status diagnosis (Christ et al., 2013). Finer norms would also suggest that the school also has concrete measures and responsibilities ready for all gradations. The categorization of norms is accompanied by a considerable loss of information, which can have a significant impact, e.g., when a child's performance falls very close to the borderline between percentiles. In this respect, combined information in the sense of positioning student performance in a percentile band with additional specification of a confidence interval is important. Within the framework of scientific research, fine norm gradations can also be processed and taken into account accordingly by means of different analysis methods. Here, a loss of information through data categorization would be detrimental. The most important question is how to design funding and resources so that children with more needs get more effective support without being stigmatized (Meijer and Watkins, 2019). The application of the norms in screening help for the application of a multilevel support system to make an important basis for the pedagogical decisions.

The following limitations of the current study must be considered. The sample used is selective and insufficiently controlled to determine the effects of compensatory measures on a school or even a classroom level. For example, the quality of the instruction at the classroom level could not be determined. Furthermore, the data collected is sufficient to model latent trajectories on a growth level but is not sufficient to model individual learning trajectories due to irregular participation. The Reiner test tends to have ceiling effects because the number of items per test is limited and only those items that correspond to the grade level spelling instruction were selected. However, this is negligible for screening purposes in the lower percentiles. Also it has to be mentioned that the test is designed as a group test with a dictation. Thus, the test is not designed to be administered individually. Another limitation of the results is the lack of comparison with an external characteristic on the basis of which the specificity and sensitivity of the results could be demonstrated over time. Differential results of Verhoeven (2000) or Lervåg and Hulme (2010) could also not be replicated due to the lack of background characteristics in this sample. For a better generalizability of the results, a sample with a higher rate of control is needed. Lastly the grade bracket of this study did not include data from first grade due to changes in the test. This shortcoming has to be addressed.

Developing screenings and progress monitoring instruments to identify children with learning difficulties is important, but not easy (Fuchs et al., 2021). The tests must be both easy to use and to interpret by teachers as well as psychometrically tested and reliable (Schurig et al., 2021). The Reiner test was constructed according to the needs of teachers and the regulations of the school system and was also able to demonstrate psychometrically appropriate goodness. A level of difficulty was selected for each grade level so that the test met the requirements of the grade level. Those grade levels aligned well across time but one should interpret the course over all 4 years only cautiously. Overall, there is considerable variation between the children, which increases rather than decreases over the years. While the test measures a more restricted test range in the first years, the test range becomes larger over the years with further requirements. Since there are fewer but still some easy items in the higher grade level test, the Reiner tests are also very sensitive to the lower percentiles.

Why are there no visible compensation effects of the tests? It has to be stated that formative assessment is still not widely used and teacher professionalism is expandable. The Reiner test concept is already in use in the inclusive region of Rügen and has proven itself as a standardized instrument with comparable norms. This makes it one of the three instruments used in Germany (Blumenthal et al., 2022). It offers a longitudinal screening with clear curricular references as well as a qualitative diagnostic that is linked to proposed pedagogical intervention. This outlines clear support structures. But in the final step, schools and sometimes even teachers in Germany decide for themselves how to deal with such offers due to the high degree of autonomy. This also includes the textbooks used and the question of the closeness to the textbooks in the design of the instruction at a classroom level. However, there is a lack of implementation in the system, training, etc. The next step for Reiner will be to examine the extent to which testing can be implemented more regularly per class and whether and where test results can be integrated into everyday school life and the associated support in learning.

## Data availability statement

The datasets presented in this study can be found in online repositories. The names of the repository/repositories and accession number(s) can be found below: OSF https://osf.io/vg2r7/, doi: 10.17605/OSF.IO/VG2R7.

## Ethics statement

The studies involving human participants were reviewed and approved by the Ministry for Education and Child Day Promotion, Germany/Mecklenburg-Western Pomerania. Written informed consent to participate in this study was provided by the participants' legal guardian/next of kin.

## Author contributions

MS did the writing and the analyses. SB provided the data and co-wrote the article. MG co-wrote the article. All authors contributed to the article and approved the submitted version.

## Footnotes

^1^ www.lernlinie.de

^2^ https://osf.io/vg2r7/
