# Supplementary material for: Continuous norming in learning progress monitoring—An example for a test in spelling from grade 2–4
Source: Front Psychol. 2022 Dec 16;13:943581. doi: 10.3389/fpsyg.2022.943581 (PMC9800992; doi:10.3389/fpsyg.2022.943581)
Supplement: Supplementary file 1 [file Table_1.docx]

**Supplement**

Supplementary Table 1: Depraved Norm Table of the Rainer

| **Grade** | **Percent solved** | **Percentile** | **lower C.I.** | **upper C.I.** | ***t* value** | **lower CI** | **upper CI** |
| --- | --- | --- | --- | --- | --- | --- | --- |
| 2 | 0 | 1.0 | 0.5 | 6.6 | 26.9 | 24.3 | 35.0 |
|  | 0.2 | 8.4 | 4.0 | 24.9 | 36.2 | 32.5 | 43.2 |
|  | 0.4 | 28.2 | 14.9 | 51.1 | 44.2 | 39.6 | 50.3 |
|  | 0.6 | 57.5 | 35.6 | 75.8 | 51.9 | 46.3 | 57.0 |
|  | 0.8 | 84.0 | 63.4 | 92.1 | 60.0 | 53.4 | 64.1 |
|  | 1 | 98.1 | 90.0 | 99.1 | 70.7 | 62.8 | 73.5 |
| 2.5 | 0 | 0.7 | 0.3 | 5.1 | 25.4 | 23.0 | 33.7 |
|  | 0.2 | 5.9 | 2.8 | 20.0 | 34.4 | 30.9 | 41.6 |
|  | 0.4 | 21.5 | 11.0 | 43.7 | 42.1 | 37.7 | 48.4 |
|  | 0.6 | 48.5 | 28.5 | 69.2 | 49.6 | 44.3 | 55.0 |
|  | 0.8 | 78.2 | 55.9 | 88.9 | 57.8 | 51.5 | 62.2 |
|  | 1 | 98.4 | 91.2 | 99.2 | 71.5 | 63.5 | 74.2 |
| 3 | 0 | 0.6 | 0.3 | 4.8 | 25.0 | 22.7 | 33.3 |
|  | 0.2 | 4.6 | 2.2 | 17.1 | 33.1 | 29.8 | 40.5 |
|  | 0.4 | 17.5 | 8.7 | 38.7 | 40.7 | 36.4 | 47.1 |
|  | 0.6 | 42.1 | 23.9 | 64.0 | 48.0 | 42.9 | 53.6 |
|  | 0.8 | 73.1 | 50.2 | 85.9 | 56.1 | 50.1 | 60.8 |
|  | 1 | 98.1 | 90.2 | 99.1 | 70.7 | 62.9 | 73.6 |
| 3.5 | 0 | 0.6 | 0.3 | 4.8 | 25.0 | 22.7 | 33.3 |
|  | 0.2 | 3.9 | 1.8 | 15.3 | 32.3 | 29.1 | 39.8 |
|  | 0.4 | 15.2 | 7.5 | 35.6 | 39.7 | 35.6 | 46.3 |
|  | 0.6 | 38.1 | 21.1 | 60.5 | 47.0 | 42.0 | 52.7 |
|  | 0.8 | 69.3 | 46.4 | 83.6 | 55.0 | 49.1 | 59.8 |
|  | 1 | 97.1 | 87.1 | 98.6 | 68.9 | 61.3 | 72.0 |
| 4 | 0 | 0.6 | 0.3 | 4.8 | 25.0 | 22.7 | 33.3 |
|  | 0.2 | 3.5 | 1.7 | 14.5 | 31.9 | 28.7 | 39.4 |
|  | 0.4 | 14.1 | 6.9 | 34.0 | 39.2 | 35.2 | 45.9 |
|  | 0.6 | 35.9 | 19.7 | 58.6 | 46.4 | 41.5 | 52.2 |
|  | 0.8 | 66.9 | 44.0 | 82.1 | 54.4 | 48.5 | 59.2 |
|  | 1 | 96.5 | 85.6 | 98.3 | 68.2 | 60.6 | 71.3 |
| 4.5 | 0 | 0.6 | 0.3 | 4.8 | 25.0 | 22.7 | 33.3 |
|  | 0.2 | 3.5 | 1.6 | 14.4 | 31.8 | 28.7 | 39.4 |
|  | 0.4 | 13.8 | 6.8 | 33.6 | 39.1 | 35.1 | 45.8 |
|  | 0.6 | 35.2 | 19.3 | 58.0 | 46.2 | 41.3 | 52.0 |
|  | 0.8 | 65.7 | 42.9 | 81.3 | 54.0 | 48.2 | 58.9 |
|  | 1 | 96.7 | 86.2 | 98.5 | 68.5 | 60.9 | 71.6 |

*Note:* The table was prepared based upon the raw scores in 0.2 intervals.
